# Reproductive Isolation in the Cryptic Species Complex of a Key Pest: Analysis of Mating and Rejection Behaviour of Onion Thrips (*Thrips tabaci* Lindeman)

**DOI:** 10.3390/biology11030396

**Published:** 2022-03-03

**Authors:** Kristóf Domonkos Király, Márta Ladányi, József Fail

**Affiliations:** 1 Institute of Plant Protection, Hungarian University of Agriculture and Life Sciences (MATE), 44 Ménesi út, H-1118 Budapest, Hungary; kiraly.kristofd@gmail.com; 2 Institute of Mathematics and Basic Science, Hungarian University of Agriculture and Life Sciences (MATE), 29-43 Villányi út, H-1118 Budapest, Hungary; ladanyi.marta@uni-mate.hu

**Keywords:** mate recognition, arrhenotoky, thelytoky, lineages, pheromone, speciation, activity

## Abstract

**Simple Summary:**

The onion thrips (*Thrips tabaci*), which is a key pest worldwide, includes three characteristic, distinct groups (i.e., lineages) under the same species name. In the current report, we addressed the question of whether individuals from these lineages recognize and assess each other as potential mating partners. We demonstrated that at least two of these lineages do not belong to the same species, since under our laboratory conditions no mating occurred between them. Moreover, specimens from these cross pairings often exhibited an escape response upon contact with the other thrips, while most of the pairs from the same lineages readily mated at their first interaction. The behaviour of males clearly indicated that they can assess the mating status of a female and usually only try to copulate with virgins. Our findings are important not only because in agriculture it is vital to know whether morphologically similar entities belong to the same species, but also because knowledge about the communication of insects and the possible role of the cues involved can help the development of new plant protection techniques.

**Abstract:**

*Thrips tabaci* Lindeman is a serious pest of various cultivated plants, with three, distinct lineages within a cryptic species complex. Despite the well-known significance of this pest, many attributes of these lineages are not yet fully understood, including their reproductive behaviour. We performed no-choice-design cross-mating experiments under a controlled laboratory environment with virgin adult individuals from all three lineages. The behaviour of thrips was recorded with a camera mounted on a stereomicroscope, and the recordings were analysed in detail. We found that the so-called leek-associated lineages of this cryptic species complex are reproductively isolated from the tobacco-associated lineage; therefore, they represent different species. Divergence in the behaviour of conspecific and heterospecific pairs became evident only after contact. There were no marked differences between the lineages in their precopulatory and copulatory behaviour, except in the duration of the latter. We confirmed mating between thelytokous females and arrhenotokous males; however, we assume some form of loss of function in the sexual traits of asexual females. The post-mating behaviour of males indicated the presence and role of an anti-aphrodisiac pheromone. We also demonstrated differences between lineages regarding their activity and their propensity for exhibiting an escape response upon interaction with heterospecific thrips.

## 1. Introduction

Cryptic species are usually defined as two or more species that are (or were in the past) classified as a single nominal species, based on their morphological similarity [1]. Many species formerly classified according to their host adaptation as generalist or polyphagous might actually be a complex of cryptic species, each with a narrower host specificity [2,3]. With the advances in molecular techniques, research on such entities has grown remarkably in recent decades [1,4], revealing cryptic species in many different animal taxa [5].

Research on cryptic species complexes is crucial for the investigation of biological diversity and for conservation purposes [1,4], but clearly such studies on arthropods could also be highly relevant to agriculture and plant protection. Recent findings show, for example, that cryptic pest species could have important differences in their host range, as is seen in *Dasineura oxycoccana* Johnson (Diptera: Cecidomyiidae) [6], or in *Bemisia tabaci* (Gennadius) (Hemiptera: Aleyrodidae) [7]. The identification of morphologically similar species with differences in their host range could also necessitate the reconsideration of quarantine actions against pests [8]. Additionally, detailed knowledge of the host adaptation of natural enemies in cryptic species complexes is vital for choosing the proper biocontrol agent in agriculture [9,10]. On the other hand, cryptic species in allopatry could represent different biological species, despite their seemingly identical host range and pest status [11]. Within a complex of cryptic pest species, an invasive species can displace the indigenous species [12,13], raising the question of whether such species displacement, which could happen undetected, could change the pest status of the insect populations present in a region.

Molecular data could indicate the presence of cryptic species in a complex; however, differences in mitochondrial DNA (mtDNA) should be interpreted cautiously, as even a deep divergence in mtDNA does not necessarily mean reproductive isolation between lineages [14,15]. Reproductive isolation could originate from either prezygotic or postzygotic barriers [16]. Since copulation, by its nature, is crucial for the production of hybrid progeny, the investigation of mating behaviour as a prezygotic barrier is clearly of great importance. Mating experiments have recently proved to be helpful for delimiting species in cryptic complexes in several insect orders, such as Hymenoptera [17], Hemiptera: Sternorrhyncha [18], Hemiptera: Heteroptera [19], and Diptera [6].

In order to mate, first the individuals of an insect pair need to be brought together from a distance, e.g., by means of volatile sex pheromones, then close range communication helps to identify each other as potential mating partners [20,21]. In this close-range recognition, cuticular hydrocarbons (CHCs) seem to play an important role in insects [22,23]; however, other types of cue might also be necessary [24,25].

The results of Pfenninger and Schwenk [5] indicate that any major metazoan taxon could possibly harbour cryptic species complexes. Therefore, it is important to study cryptic complexes in all animal taxa, particularly those orders that comprise species that are usually hard to detect or identify, such as Thysanoptera (thrips). The order Thysanoptera comprises tiny insects, usually only 1–2 mm long, whose morphological identification calls for careful work and high expertise. Many species of thrips are serious pests in a wide range of plants with economic importance, causing damage to them either directly through feeding, or indirectly by the transmission of orthotospoviruses [26,27,28]. Most of the pest thrips species belong to the family Thripidae [27], a taxon with more than 2000 species [29]; however, recent studies showed that many of these important thrips pest species are likely a complex of cryptic species, including *Frankliniella occidentalis* (Pergande) [30], *Scirtothrips aurantii* (Faure) [8], *Scirtothrips dorsalis* Hood [31], *Frankliniella schultzei* Trybom [32] and *Thrips palmi* Karny [33].

Aside from the aim of testing reproductive isolation between (cryptic) species, knowledge of the mating system and behaviour of insect pests is also crucial to control and manage them effectively [34,35,36]. However, many elements of the reproductive biology of most thripid pest species are still unknown.

Thrips are usually considered to be haplodiploid, and both arrhenotoky and thelytoky are common types of reproduction among them. In the case of arrhenotoky, fertilized eggs produce diploid females with two sets of chromosomes (one of maternal origin and the other of paternal origin), whereas unfertilized eggs produce haploid males with a single set of maternal chromosomes [37,38]. Thelytoky (obligate parthenogenesis) in Thysanoptera is automictic, and the ploidy restoration results in only female progeny [37,38]. In nature, some thripid species are known to form male aggregations, where they also mate with the landing females [39,40,41]. The formation of these aggregations is probably mediated by male-produced aggregation pheromones [42].

A detailed description, with some additional notes on other species, of the mating behaviour is available for only five species: *Frankliniella occidentalis* (Pergande) [43,44], *Frankliniella schultzei* Trybom [45], *Scirtothrips aurantii* (Faure) [46], *Echinothrips americanus* Morgan [47] and *Megalurothrips sjostedti* (Trybom) [41]. However, in the case of cryptic complexes, these descriptions might not be perfectly correct for all the species or lineages.

Species in the family Thripidae seem to differ in the number of matings during their lifetimes. Males are able to inseminate many females [43,47], but they can also be choosy and avoid mating [41]. In some species, females only mate once—or at least refuse to mate for an extended period of time after one successful mating—which is probably the result of a male-produced anti-aphrodisiac pheromone applied on the back of the females during mating [44,48,49]. In some other species, however, females mate multiple times [41,50].

The onion thrips (*Thrips tabaci* Lindeman) was one of the first thrips species suspected of being a complex of cryptic species [51]. However, *T. tabaci* is usually considered as a single cosmopolitan, major pest species of various plants, including cabbage, onion and tobacco [52,53,54,55], and also an important vector of plant pathogenic viruses in the genus *Orthotospovirus* (Tospoviridae) [28,56].

The presence of a cryptic species complex was first proposed by Zawirska [51], who recognized two “biological types” within the complex, with well-characterized differences in the biological and ecological traits of these types. Several decades later, after investigation of sequence variation of the mitochondrial cytochrome oxidase I (mtCOI) gene, Brunner et al. [57] described three distinct groups within the onion thrips species complex, and named them L1, L2 (leek-associated) and T (tobacco-associated) “lineages”.

To avoid further confusion, we shall here use the term “lineage” rather than the criticised term “biotype” [58].

Further findings support distinct lineages and confirm that the T and the L1 lineages reproduce by arrhenotoky, while the L2 lineage reproduces by thelytoky [59,60,61,62,63,64]. However, most of these studies focused on mtDNA for separation, and no efforts were made to screen for reproductive isolation. Despite the genetic divergence, differences in morphological characteristics that could be used for the identification of these lineages are not known among adult onion thrips individuals (hence, they are cryptic).

While it is difficult to determine the exact host range of these lineages (mainly because of the lack of identification of the lineages in many papers, together with other differences such as variation in the definitions used), we think that the L1 and L2 lineages could be considered as polyphagous, whereas the T lineage has a narrower host range (reviewed in [65,66]). It seems well established that the L1 and L2 lineages can occur in sympatric populations, and both occur and breed well on cabbage and *Allium* plants [57,67,68], while the T lineage has a pronounced preference for tobacco [51,57], a plant on which the L lineages fail to survive [69]. Note, that while all three *T. tabaci* lineages can be found in Europe [57,64], it seems that the T lineage is absent in the USA [70]. While the other two lineages can also transmit it, the T lineage is the most efficient vector of *Tomato spotted wilt virus* (Tospoviridae), which is probably the most important plant pathogen transmitted by the onion thrips [51,69,71,72].

In onion thrips, male aggregations have not been reported, no pheromone substances have been identified, and there is no available, detailed description of the mating behaviour. Moreover, the reproductive isolation between the lineages has only been investigated among L1 and L2 lineages [73]. This study revealed that L1 males readily mate with both L1 and L2 females and show no preference for females from either lineage. A low rate (ca 1.9%) of gene transfer was also detected in the F_1_ generation, after successful copulations of L2 and L1 *T. tabaci* individuals [73]. This is in contrast with an earlier claim—on the basis of microsatellite analysis at nine loci—that sexual and asexual onion thrips lineages are genetically isolated [74].

The aim of this paper was to investigate the possibility of reproductive isolation within the *T. tabaci* species complex, to gain detailed insight into the behaviour of thrips, and to test for differences within and between lineages. Therefore, we performed cross-mating experiments with all the three known onion thrips lineages, and analysed the thrips’ behaviour from video recordings.

Here, we present detailed comparative data for the copulatory, pre- and post-copulatory behaviour of *T. tabaci* lineages that mate with each other, together with information on the behaviour of lineages that are reproductively isolated. Our findings also help to improve the knowledge of communication in onion thrips and the possible roles of pheromones.

## 2. Materials and Methods

### 2.1. Insect Cultures and Rearing

Stock cultures of the three distinct *T. tabaci* lineages (T, L1 and L2) were kept separately and maintained at room temperature at the Hungarian University of Agriculture and Life Sciences, Hungary. Thrips in the cultures were reared on leaves of tobacco (T lineage), leek (L1 lineage) or cabbage (L2 lineage) in ventilated plastic or glass containers. For details about the establishment of these cultures, see Farkas et al. [64].

In order to have thrips with a known pedigree, isofemale lines were established. Randomly selected adult females (“mothers”) from the stock cultures were placed individually into microcentrifuge tubes (2 mL) with leaf discs of tobacco (T) or cabbage (L1 and L2 lineages). Based on the known host range and occurrence of the lineages, these food sources represented natural conditions. The leaves were checked beforehand using the bottom light of a stereomicroscope, and leaf discs with thrips eggs or feeding scars were discarded. Females from the arrhenotokous cultures had usually mated already and produced more female than male offspring; therefore, to obtain a sufficient number of male progenies from the arrhenotokous biotypes, we also separated some juvenile thrips, as they later developed into virgin mothers and produced 100% male progeny. New leaf discs were provided regularly for the individually isolated mothers. After the hatching of eggs oviposited into the leaf discs by either mated or virgin females, larvae were separated individually into new microcentrifuge tubes (2 mL) with leaf discs of tobacco (T) or cabbage (L1 and L2 lineages) to ensure their virginity as adults. The tubes were monitored regularly (daily from the pupal stage) for adult emergence to obtain adults with a known age. The sex of the emerged adults was determined by the known differences between sexes [26]. All the microcentrifuge tubes with thrips were kept in a climate chamber at 23.5 ± 1 °C with a photoperiod of 16:8 h (light:dark).

All of the isofemale lines used in this study were identified to *T. tabaci* lineage level based on the molecular method of Farkas et al. [64]. For the identification, either the mothers or their progeny were used. Briefly, first, total genomic DNA was extracted from thrips of every isofemale line. Then, specific forward (5′-ATTAATTATAGGRCTTTAYAAAGAAGG-3′) and reverse (5′-GTAGTGAAAGTGAGCTACAACATAATAAGT-3′) primers were used for PCR amplification of a mitochondrial COI (mtCOI) fragment of 780 bp. After amplification, the PCR products were digested with PsuI and PsyI restriction enzymes. Using this method, the *T. tabaci* lineages (T, L1 and L2) can be unambiguously sequestered by gel electrophoresis, since the L1 lineage has restriction sites for both endonucleases, which results in three different sized fragments (345 bp, 274 bp and 161 bp long), the L2 lineage has only one restriction site; therefore, digestion produces two fragments (619 bp and 161 bp long), and the T lineage does not have restriction sites, thus it remains in one 780 bp long fragment [64].

One isofemale line, which had an mtCOI not characteristic of its lineage, was excluded from the statistical analysis.

### 2.2. Cross-Mating Experiments

No-choice cross-mating experiments were carried out among the three known *T. tabaci* lineages. The existence of two arrhenotokous (T and L1) lineages and one thelytokous (L2) lineage in the species complex resulted in six possible combinations of males and females. T♀ + T♂ and L1♀ + L1♂ pairings were considered as primary, and L2♀ + L1♂ pairings as secondary controls, because Li et al. [73] reported that L2 females mate with L1 males, but this had to be verified with our populations. The remaining three combinations were called cross pairings.

For the tests, a virgin male and virgin female, 2–7 days after adult eclosion, were placed together in the cap of a 2 mL microcentrifuge tube, covered with a glass cover slip. A new arena (approximate diameter of 8.5 mm) was used for every pair. In total, the behaviour of 234 pairs (32–45 replicates from each of the possible six combinations) were investigated. Thrips were handled with fine brushes and disturbed as little as possible (usually only a few seconds). The mating experiments were conducted from October 2017 until February 2018, under room temperature, within the daylight photoperiod of the insects, in a laboratory room illuminated with white artificial or natural light, or both. The behaviour of the specimens was monitored and recorded for 10 min without disturbance with a Sony XCD-SX90CR digital camera (Sony, Tokyo, Japan) mounted on a Zeiss Stemi 2000-C stereomicroscope (Zeiss, Oberkochen, Germany) with illumination from a Schott KL 200 LED light source (SCHOTT AG, Mainz, Germany), which emitted white light. Recordings started immediately after placing the insects into the arena, and the cover slip on the top of it. Recordings could be longer if an interaction event, precopulation or copulation had already started but was not finished within 10 min. In some cases, when in a cross pairing, the specimens did not successfully mate with each other, the thrips were used once again in a control pairing with another thrips from the opposite sex, to check whether or not their earlier behaviour was only the result of an error in recognising each other as potential mating partners.

Video playbacks have been used for the extensive investigation and analysis of specific behaviours. Since the same person performed the bioassays and investigated the video recordings, it was not possible to record the data blind. In all of the combinations, the primary focus of the mating tests was which steps, along the mating behaviour sequence, were completed by the observed pair. For the mating behaviour sequence, we used the same behavioural traits as Akinyemi and Kirk [44] with slight modifications, as introduced in Table 1. We also recorded whether remating occurred during the observation period.

For each pair, the time until the first interaction event and the sex of the mate that approached the other in the first interaction event were also recorded. We scanned for the so-called “interaction events” in our video recordings, as our preliminary observations showed that while thrips did not instantly detect each other when placed together into the cap of a microcentrifuge tube, they had to be quite close (approximately 1–2 mm), but not necessarily touching each other, to show any specific changes in behaviour. If male mounting occurred, then the time between first contact and first male mounting was also determined. More detailed description of the monitored behaviours is given in Table 1 and Table 2.

### 2.3. Behaviour of Mating Pairs and Post-Mating Interactions

Mating and precopulatory behaviour was visually analysed from 69 mating pairs, with the replaying of the video recordings multiple times, to ensure that behaviours were correctly defined and timed. Investigation focused on whether the pair mated in their first interaction event, the duration of precopulation and copulation, the steps of the mating behaviour sequence, positions and the general behaviour of the female and male—including antennation and stroking. For the precopulation, we also recorded which thrips approached the other, the first contact position, and whether female rejection and male identification behaviour occurred during precopulation (Table 2). We measured the duration of precopulation with three different starting points: from the beginning of the interaction event that led to successful mating (“Precopulation 1”), from the first contact of the interaction event that led to successful mating (“Precopulation 2”), and from the male mounting that led to successful mating (“Precopulation 3”). The end of the precopulation was the moment when the male inserted his aedeagus into the female genitalia, from which successful copulation started.

The interaction events after successful matings within the 10-min-long observation period were also investigated to check for differences in behaviour before and after copulations. The mating happened at the latest during the pairs’ sixth interaction event; therefore, we used the first 6 interaction events after the copulation for the before/after comparison. We recorded whether in a given interaction event male mounting occurred, and if so, whether male or female (or both) rejection behaviour occurred after that. Then, ratios of interaction events with these behaviours were calculated before and after copulations as follows: (1) the number of interaction events when male mounting occurred divided by the total number of interaction events; and (2) the number of interaction events when male and/or female rejection occurred after male mounting divided by the number of interaction events when male mounting occurred.

### 2.4. Behaviour of Non-Mating Cross Pairs

When no successful mating occurred in the cross pairings, the total number of interaction events up to 10 were considered during the whole 10-min-long recording period.

If no mating took place, then we also determined how the specimens departed from each other at the end of each interaction event. Observed behaviours were grouped into two categories. A thrips either exhibited an escape response (see Table 2 for description, and Videos S4 and S5 for examples) or remained more or less calm (standing still, just walking/jogging away, or sometimes showing some searching behaviour). The grouping was irrespective of whether mating attempts were rejected earlier. For the analysis of this behaviour, we only used the first 6 interaction events within each recording, since it was rare to have more than six interaction events, and also because our results showed that the mating pairs mated in their sixth interaction event at the latest.

### 2.5. Statistical Analysis

Statistical analysis was performed with statistical software IBM SPSS (v25.0, Armonk, NY, USA [75]) and Microsoft Excel v2019 [76].

In the control pairings, there were cases when one or both specimens from the pair were used earlier in a cross pairing (see: Section 2.2); therefore, we examined whether pairings in which both of the specimens were used for the first time differed from those in which one or both specimens were used for the second time. For the comparison between these two groups, three Fisher’s exact tests were run, each for 2 × 2 contingency tables, with the number of pairings that (1) interacted or not; (2) successfully mated (considering the total number of pairings) or not; and (3) successfully mated (considering only those pairings, in which interaction occurred) or not. No significant differences were found (Fisher’s exact tests, *p* > 0.05; Table 3). Therefore, we concluded, that the exposure to another adult thrips without successful mating did not influence the behaviour of thrips, and thus these data were pooled together.

#### 2.5.1. Cross-Mating Experiments

For the cross-mating experiments, the fixed steps in mating behaviour were analysed with Fisher’s exact tests for 6 × 2 contingency tables with the number of pairings that did or did not complete that step in the mating behaviour as one factor, and the six different pairings as the other. Replication numbers were always calculated as the number of pairings that completed the previous step. For the analysis of the frequency of successful mating, the same test was used but with a 3 × 2 contingency table; cross pairings were excluded because of the low number of replicates. When there was a significant overall difference between the pairings, Marascuilo’s procedure [77] was used to compare the percentage of pairs that completed a step in the mating behaviour sequence.

The time until the first interaction event, and the time between first contact and first male mounting were analysed by one-way ANOVAs, after an ln(x + 1) transformation. Prior to analysis, the normality of the residuals was checked by their skewness and kurtosis according to Tabachnick and Fidell [78]. The homogeneity of variance assumption was tested with Levene’s tests, and confirmed in the case of time until first interaction event (F_(5,167)_ = 0.642, *p* = 0.668), but rejected in the case of time between first contact and first male mounting (F_(5,89)_ = 2.923, *p* = 0.017). Therefore, post hoc comparisons used the Games-Howell test.

#### 2.5.2. Which Sex Approached, Precopulation and Copulation

McNemar’s tests were used for analysing which thrips approached the other one during the first interaction event and during the interaction event that led to successful mating (precopulation). Three categories were used and the frequencies of cases were compared within each pairing: male approached the female, female approached the male, or both were approaching each other (i.e., moving in the direction of the other). Cases with unclear situations were excluded from the analysis (for example, if thrips were already close to each other at the start of the video recording). For this behaviour in the precopulation, a Fisher’s exact test was run for a 3 × 3 contingency table to check for overall differences across pairings. Adjusted standardized residuals were also calculated and identified. Their squared values follow a chi-square distribution with 1 degree of freedom, so adjusted standardized residuals above 1.96 in absolute value indicate significant over- or underrepresentation, depending on their positive or negative signs.

The frequencies of the different types of first contact positions at precopulation within each pairing were compared with a McNemar’s test.

To compare the behaviour of control pairs during precopulation, Fisher’s exact tests were run for 3 × 2 contingency tables to analyse the frequencies of pairs that mated in their first or for a later interaction event, the frequencies of pairs with female rejection behaviour and the frequencies of pairs with male identification behaviour. Adjusted standardized residuals were calculated and identified when relevant (i.e., in significant cases). Cases where the male identification behaviour was unclear were excluded from the analysis. One-sample z-tests were run for the male identification to check if the occurrence of this behaviour was by chance or consistent.

Durations of precopulation and copulation were analysed with one-way MANOVA tests, after ln transformation. Prior to analysis, 4 extreme cases were excluded since the duration of copulation of one T♀ + T♂ and two L1♀ + L1♂ pairs, and the duration of precopulation of one L2♀ + L1♂ pair was extremely prolonged. Given the significance of the overall test, the univariate main effects were examined with Bonferroni’s correction. Normality of the residuals was checked by their skewness and kurtosis according to Tabachnick and Fidell [78]. The homogeneity of variance assumption was tested with Levene’s tests, and confirmed in the case of duration of Precopulation 1 (F_(2,60)_ = 0.008, *p* = 0.992), Precopulation 2 (F_(2,60)_ = 0.403, *p* = 0.670), and Precopulation 3 (F_(2,60)_ = 0.401, *p* = 0.671) but rejected in the case of duration of copulation (F_(2,60)_ = 4.596, *p* = 0.014). Therefore, post hoc comparisons used the Games-Howell test. For the behaviours and durations of precopulation and copulation, only the first mating of each pair was used in statistical analysis, and rematings were excluded.

#### 2.5.3. Post-Mating Interactions

To detect whether the ratios of interaction events with male mounting and male or female rejection changed before and after copulation, we ran a paired Wilcoxon’s signed rank test as it does not require any assumption for the distribution, thus we could use it even if we had some scarce and not normally distributed data.

#### 2.5.4. Non-Mating Pairs

For the analysis of the total number of interaction events across the cross pairings, each replicate was allocated to one of five categories. 1: no interaction event occurred during the observation period; 2: one interaction event occurred; 3: two to six interaction events occurred (six interaction events were chosen as a limit, because mating pairs mated at the latest in their sixth interaction event); 4: seven to nine interaction events occurred; 5: ten or more interaction events occurred. After this, a Fisher’s exact test was run for a 5 × 3 contingency table with the calculation and identification of adjusted standardized residuals.

To analyse the frequencies of escape responses at the end of each interaction event, we first checked whether the frequency of this behaviour changed across the first six interaction events within each of the cross combinations. For this, Fisher’s exact tests were used in 6 × 2 contingency tables with the number of pairings in which the female exhibited an escape response or not at the end of the interaction event as one factor, and the interaction events from number 1 to number 6 as the other. We analysed the behaviour separately for males and females. With the three different cross pairings for females and males separately, we ran six Fisher’s exact tests, and none of them gave a significant result (*p* = 0.627 and *p* = 0.962 for T♀ + L1♂ pairings, *p* = 0.701 and *p* = 0.699 for L1♀ + T♂ pairings, *p* = 0.863 and *p* = 0.124 for L2♀ + T♂ pairings for females and males, respectively). Because of this, we pooled the data from the first six interaction events together and compared the frequencies of escape responses across the three cross combinations for females and males, separately. Again, Fisher’s exact tests were run, now for 3 × 2 contingency tables with the number of interaction events with or without escape responses (by female or male separately) at the end as one factor, and the three cross combinations as the other. Adjusted standardized residuals were also calculated and identified.

## 3. Results

### 3.1. Thrips tabaci Lineage Identification

In total, 113 isofemale lines were used in the experiment, 43 of which originated from our T, 46 from our L1 and 24 from our L2 stock cultures. Based on their mtCOI region, all of the lines proved to belong to the lineage as expected, except one. This one isofemale line originated from our L1 culture, and the mtCOI of one adult and one juvenile thrips was investigated. The digestion with the endonucleases resulted in two fragments only, displaying mtCOI characteristic of the thelytokous lineage; however, the isofemale line was found to be arrhenotokous, as all the offspring that reached adulthood were males.

### 3.2. Cross-Mating Experiments

In total, 234 video recordings were used in our analysis with pairs from the six combinations of virgin female and virgin male specimens belonging to different *T. tabaci* lineages. In 173 of these recordings, interaction events were observed (Table 4). During our 10-min-long standard observation period, no successful copulations occurred in T♀ + L1♂ or L1♀ + T♂ combinations and only two matings were seen between L2♀ + T♂ pairings (Table 4). On the other hand, considering the total number of pairings, T♀ + T♂, L1♀ + L1♂ and L2♀ + L1♂ combinations resulted in successful matings in 52%, 70% and 54% of cases, respectively. We found no significant difference between our primary and secondary control pairings (Fisher’s exact tests and Marascuilo’s procedure, *p* > 0.05; Table 4); therefore, from now on they will both be referred to as control pairings.

A difference between the control and cross pairings was apparent after Phase 2 (Contact), when T males avoided mounting L1 or L2 females, and L1 males avoided mounting T females; this difference in behaviour was statistically significant compared to the control combinations (Fisher’s exact test, *p* < 0.001 and Marascuilo’s procedure, *p* < 0.05). The divergence continued in the next phase, as the difference was also statistically significant between crosses and control pairings in the frequency of pairs with males, not just mounting the females but also bending their abdomens beneath those of the females (Fisher’s exact test, *p* < 0.001 and Marascuilo’s procedure, *p* < 0.05; Table 4).

After successful mating, five pairs—3 from T♀ + T♂ and 2 from L1♀ + L1♂ combinations—mated for a second time during the 10-min-long observation period.

The mean time from the beginning of the recording until the first interaction event was not significantly different across the pairings (F_(5,167)_ = 0.630, *p* = 0.677), but the time intervals males needed to mount the females, counting from the first contact, were significantly different across the pairings (F_(5,89)_ = 12.014, *p* < 0.001). Males needed less time to mount the females in the control pairings than in the crosses, but the difference was not significant in all control vs. cross pairwise comparisons (Games-Howell, *p* > 0.05; Table 5).

We found notable differences regarding which sex approached the other during the first interaction event (Table 6). With the exception of the L1♀ + L1♂ combination, in all the pairings, either the males or the females were significantly more active than the opposite sex in this regard, and the rarest case was when both the male and the female were moving towards the other. In the control pairings, either the males were more active than the females (T♀ + T♂ and L2♀ + L1♂ combinations, McNemar’s test, *p* < 0.05 and *p* < 0.01, respectively), or there was no significant difference between the two sexes (L1♀ + L1♂ combination, McNemar’s test, *p* = 0.824). In the cross pairings, irrespective of sex, it was always the T lineage that had significantly less frequent activity (i.e., approaching the other individual fewer times, Table 6).

### 3.3. Precopulation and Copulation Behaviour

From the observed 69 matings, 67 happened in control pairings. In the remaining two cases, L2 lineage females mated with T lineage males. Due to this low replication number, these two cases were not included in statistical analysis, and mating behaviour was identified from the first successful matings of control pairs (see Videos S1–S3 for examples).

Precopulatory and copulatory behaviour of *T. tabaci* pairs followed a general pattern, but not every behaviour element of this general pattern was detectable in all pairs. We found no seemingly important differences among the control pairings in this regard; therefore, we are going to discuss and show their behaviours pooled together, if not stated otherwise.

We were not able to detect any specific behaviour before interaction events that could have had a connection with successful mating, such as increased activity or vibration of wings, as was found by Milne et al. [45] with *F. schultzei*. However, the precopulation and mating behaviour of *T. tabaci* generally followed the same sequences as of other reported thripid species [41,43,44,45,46,47].

From the mated control pairs, 20 T♀ + T♂ pairs (*n* = 22), 15 L1♀ + L1♂ pairs (*n* = 23) and 15 L2♀ + L1♂ (*n* = 22) pairs mated in their first interaction event. The distributions of those pairs that mated in their first or in a later interaction event were not significantly different across control pairings according to the Fisher’s exact test (*p* = 0.097). From the remaining control pairs, nine mated in their second, one in their third, four in their fourth and three in their sixth interaction event.

In the interaction events that led to successful mating in the control pairings, males approached the females more often than vice versa; however, the difference was only significant in the case of T♀ + T♂ and L2♀ + L1♂ combinations (McNemar’s test, *p* < 0.05; Table 7). Fisher’s exact test was not significant (*p* = 0.250), but the adjusted standardized residual was 2.2 in the L1♀ + L1♂ combination for the female approaching, indicating a slightly significant overrepresentation in that case only.

After approaching each other, at the start of the precopulation, males typically displayed a so-called “identification behaviour”. This behaviour usually happened before the first contact when the male was moving close to the female. It was characterized by a sudden halt in all movements of the male, including walking or running and moving the antennae, which were usually held fixed and more or less high. This pause in movements was occasionally very brief (<1 s), but usually lasted a few seconds, while no or only very little movement occurred. Usually, at the end of this phase the male stepped closer, made contact with the female, and started to mount her. Statistical analysis confirmed that the frequency of occurrence of this behaviour is not different across pairings (Fisher’s exact test, *p* = 0.322) and that it is a consistent part of the males’ behaviour (one-sample z-tests, *p* < 0.05; Table 8).

During precopulation, the thrips first contacted each other usually either in a head-to-head position, or the male touched the female’s thorax or abdomen with his antennae. The female head to male thorax or abdomen position was less frequent in every combination, but the difference was significant only in the T♀ + T♂ pairings (McNemar’s test, *p* < 0.001; Table 9).

After physical contact, male mounting followed as the next step in the mating behaviour sequence. Sometimes a male contacted a female more than once before successful mounting, but these contacts usually followed each other quickly, and actual separation of the insects was rare. A male could successfully climb onto the back of a female from either the front, from the back or from the side. Upon mounting the female, the male aligned his body (when it was necessary) to face the same direction as the female, then bent his abdomen beneath that of the female. Next, the male inserted his aedeagus into the female genitalia and copulation started. These elements usually followed each other in smooth rapid succession in a few seconds, but occasionally males had to repeat either the mounting, the alignment of their body, or the twist of their abdomen to find correct position before successful intromission.

During the precopulation, the females were usually calm, but often took a few steps and sometimes antennated. Escape responses or escalated female rejection were rare, but some types of rejection (arching and/or flipping their abdomen up and down), often mild, occurred surprisingly frequently (Table 10). The overall difference in the distribution of rejection by the females across the control pairings was not significant (Fisher’s exact test, *p* = 0.090), but rejection occurred most often in the L2♀ + L1♂ combination. Absolute values of the adjusted standardized residuals were above 1.96 in the L2♀ + L1♂ combination, and close to it in the L1♀ + L1♂ combination, indicating a slightly significant overrepresentation of rejection in the former, and underrepresentation of rejection in the latter combination (Table 10).

As a male climbed onto her, a receptive female usually raised the end of her abdomen slightly as was seen in *Frankliniella occidentalis* [44]. This could serve as a help for the insertion of the male’s aedeagus, but this was not evident on every occasion.

Regarding the movement of the males’ antennae (antennation) and stroking with the legs during precopulation and copulation—which seems to be an important and characteristic part of the thrips’ mating behaviour—our observations showed the following. Typically, upon successful mounting and the alignment of his body, a male immediately started antennating and stroking the female’s back with one of his mesothoracic legs in a very excited manner. This phase of excitement usually ended with successful intromission, either straight away or a few seconds later. During this time, both the antennae and the mesothoracic leg were moving fast and with a big range of motion, with his antennae moving up and down. If a male had to try bending his abdomen beneath that of the female more than once, movements of the antennae and legs were usually paused between attempts. The phase of such excitement sometimes ended quite suddenly and sometimes slowed down gradually. Either way, during the main part of the copulation, both antennation and stroking continued, but with an overall deceleration and calming down during copulation, as movements usually slowed down a bit and became less frequent. Pauses in movements also appeared, although the movements sometimes became faster again. The antennation with large up and down movements usually changed to a much shallower and more irregular movement and vibration. Pauses in antennation and stroking happened at the same time or separately from each other. Longer breaks in movements seemed to become more frequent in the second part of the copulation.

During copulation, although males often used just one mesothoracic leg for stroking the female’s back, movements of other legs were also observed, but less frequently. The possible role of these movements could be to secure the position of the male during mating.

During copulation, in almost all cases, the male remained on the back of the female or slid slightly onto her side. The so-called “V-position” (which appears to be part of the mating behaviour of some other thripid species [41,44,45]) was observed in only four cases, of which in two cases the male again climbed onto the back of the female before copulation ended. However, we frequently observed during mating, especially in the first part of copulation, that the head and the body of males occasionally rose higher for a moment; sometimes this was just the head and body, but from time to time, even the front legs left the females’ backs. We assume that this behaviour could aim to secure a male’s position, but in some cases it could lead to falling off from the back from the female, leading to a V-shape position.

Females were usually calm and standing still throughout the main part of the mating. Only some females made a few steps occasionally, or antennated, but rejection behaviour or an escape response was quite rare.

Just prior to separating, the movements of the male usually increased a little. This was usually the movement of the legs and stroking, but an increase in antennation was also frequent. Females usually stayed calm throughout the last part of the mating as well, but the frequency of movements (such as steps and antennation but also trying to run away) increased. However, these behaviours only rarely contributed to separation as copulation usually ended with the male climbing down from the back of the female and pulling the tip of his abdomen apart from hers.

Regarding the duration of precopulation and copulation, the one-way MANOVA revealed a significant multivariate main effect of pairing (Wilks’ λ = 0.534, F_(8,114)_ = 5.246, *p* < 0.001). The univariate main effects showed no significant differences among the control pairings for the duration of precopulation (Table 11). However, the duration of copulation was significantly different, and pairwise comparisons revealed that all control pairings differed from each other; the mating of L1♀ + L1♂ pairs was the shortest, and T♀ + T♂ was the longest.

The duration of copulation of the three pairs that were excluded from the statistical analysis were 465, 324 and 395 s for the two L1♀ + L1♂ pairs and one T♀ + T♂ pair, respectively. Despite their prolonged mating, there was no clear deviation in their behaviour from the general pattern described earlier. The duration of precopulation of the L2♀ + L1♂ pair that was also excluded from the analysis was 251 s from the beginning (and from the first contact) of the interaction event. During this time, the male continuously tried to mate with the female, but she constantly rejected him. The number of male mounting and abdomen twisting attempts was more than 20. Finally, though, the pair copulated successfully for 128 s.

The rematings, observed in only five pairs in total, did not differ in general from the first matings regarding the behaviour of the insects, although the duration of these matings was only 59, 71 and 82 s for the three T♀ + T♂ pairs, and 104 and 156 s for the two L1♀ + L1♂ pairs, respectively.

The behaviour of the two L2♀ + T♂ cross pairs that mated also seemed to fit into the usual pattern observed with the control pairings; however, the durations of copulations were notably shorter in these cases as well: 71 and 70 s. The first pair mated in their first interaction event, but with a rather longer duration of precopulation (24 s from the beginning of the interaction event and from the first contact, and 12 s from male mounting), during which the female at first tried to reject the male. The second pair mated in their third interaction event, but with a really short precopulation (3 s from the beginning of the interaction event).

### 3.4. Post-Mating Interactions

Based on our observations, it seemed that the behaviour of the insects became calmer after the copulation, but this was not possible to quantify. Therefore, we only aimed to focus on whether their behaviour changed regarding the recognition of each other as suitable mating partners.

Upon completing the copulation, in most cases the insects did not walk or run too far away from each other but stayed close and soon engaged in a new interaction event. Sometimes, though, either the male, the female or both of them left the area after successful copulation. However, irrespective of how far they moved from each other after copulation, at the beginning of the upcoming interaction events, mated thrips seemed to behave the same way as a virgin pair. After copulation, the ratio of interaction events with male mounting decreased, compared to the interaction events before copulation, but this was only significant in the case of T♀ + T♂ and L1♀ + L1♂ combinations (Table 12). Moreover, there was a huge increase in the ratio of interaction events with male rejection behaviour, which was significant in every pairing (*p* < 0.001; Table 12). Before copulation this behaviour was only observed at one T♀ + T♂, and one L2♀ + L1♂ pair. After copulation, males displayed this behaviour in 76–88% of the interaction events, when male mounting occurred. During male rejection behaviour, males sometimes started to mount the females, then stopped and reversed the movement, but sometimes they climbed successfully onto the females’ backs and could even bend their abdomens beneath those of the females, before they discontinued the mating sequence, stopped trying to mate with the females, and climbed down from their backs (see Video S7 for an example of male rejection behaviour). Male rejection behaviour sometimes occurred together with female rejection, but cases were only counted as male rejection behaviour when a male climbed down from the back of a female, rather than falling off.

After copulation, there was also an increase in the ratio of interaction events with female rejection behaviour after male mounting, but this was only significant in the case of T♀ + T♂ pairings (*p* < 0.001; Table 12). These results support the conjecture that, though males often start to try and copulate again with the females they have mated with before, they eventually sense something that forces them to avoid proceeding further in the mating sequence, and this is not simply because of the rejection behaviour of the females.

Before copulation, the ratios of interaction events with male mounting were 1.00 and 0.67 for the two mating L2♀ + T♂ cross pairs, but none of the males mounted the females again during the upcoming six and four interaction events after the copulation.

### 3.5. Behaviour of Non-Mating Pairs

Fisher’s exact test showed a significant overall difference in the distribution of the total number of interaction events across the different cross pairings (*p* < 0.05). The calculation and investigation of the adjusted standardized residuals revealed that the difference is pronounced between T♀ + L1♂ and L1♀ + T♂ combinations. In the T♀ + L1♂ combination, no interaction event (Category 1) was significantly overrepresented, while in the L1♀ + T♂ combinations, the significantly overrepresented frequency was Category 5 (10 or more interaction events) (Table 13). Meanwhile, the significantly underrepresented categories of T♀ + L1♂ and L1♀ + T♂ were Category 5 and Category 1, respectively.

We observed that escape responses occurred surprisingly often at the end of interaction events, and Fisher’s exact tests showed significant distribution differences between combinations for these responses (*p* < 0.001; Table 14). The adjusted standardized residuals revealed that the differences are again pronounced between T♀ + L1♂ and L1♀ + T♂ combinations. T lineage onion thrips reacted with an escape response significantly more often than L1 lineage onion thrips, irrespective of the investigated sex (Table 14). Interaction events with or without escape responses at the end of them are shown in Videos S4–S6 as examples. 

## 4. Discussion

### 4.1. Reproductive Isolation in the Onion Thrips Cryptic Species Complex

In our cross-mating experiments with a 10-min-long observation period, no successful copulations occurred between pairs of males and females of the two arrhenotokous *T. tabaci* lineages (T and L1) out of 45 and 32 pairings (T♀ + L1♂ and L1♀ + T♂ combinations, respectively), despite the fact that the majority of these pairs interacted and contacted each other. On the other hand, if interaction occurred, almost all pairs from the T♀ + T♂ and L1♀ + L1♂ combinations mated, resulting in successful copulations in 52% and 70% of the total number of pairings.

The mating behaviour sequence of L2♀ + T♂ pairs was almost identical to that of the other two cross combinations (T♀ + L1♂ and L1♀ + T♂), with the exception of Phase 5 (successful mating), since two males from the T lineage eventually copulated with thelytokous females. However, taking the total number of cross pairings together (118 cases), we found a rather low number of matings among these pairs (2 cases), despite the following. First, in our experimental setup, any long-distance cues, which would bring the individuals close to each other in nature, were prevented by the confinement of the thrips in a small mating arena, and this could have increased the chance of heterospecific mating [20]. Rafter and Walter [46] found for example that, under laboratory conditions, 9.1–15% of the *Scirtothrips aurantii* cross pairings from different populations mated, despite molecular investigations showing that there was a lack of gene flow across these populations and that they are different species [8]. Additionally, we only used individually reared virgin males and females, and virgin male thrips could be less discriminating among females, as was pointed out by Akinyemi and Kirk [44] in the case of western flower thrips (*F. occidentalis*). Moreover, males should be less choosy in systems where for peak female fitness only a small number of matings is needed [79]. In the *T. tabaci* cryptic species complex we only have data for the mating frequency of the L1 lineage, where females mated on average 2.3 times during a 30-day period (which is close to their mean longevity), but approximately 20–30% of the females only mated once [50]. We can still treat this as a low female mating frequency, considering that, despite prospermatogeny, males of a thripid species could inseminate up to 10 females [47].

Mating frequency was very low in the cross pairings, but significant difference was not detected in the frequency of pairs that completed Phase 2 (Contact) across all combinations. The divergence became evident in the next phase, when substantially lower numbers of males mounted females in the cross pairings. However, the frequency was not zero; 17–36% of the cross pairs completed this phase, although on average it took more time for them than for the control pairs. The frequency of pairs where bending of the abdomen was completed was also significantly different, with a very rare occurrence in cross pairings. These results corroborate the findings of Miller et al. [80] made on paper wasps, where males were equally likely to inspect conspecific and heterospecific females, although they were slower to inspect the latter, and male mounting was already significantly more frequent in conspecific pairs. Puniamoorthy [81] observed a similar pattern with a dung fly, where in one-on-one trials, no mating occurred if the male and female were from different populations, although males sometimes made attempts to mount the females, but the frequency of these attempts was lower than if the individuals were from the same population.

While two L2♀ + T♂ cross pairs in our experiments eventually mated, and there were no observable deviations in their behaviour, the durations of their copulation were considerably shorter (71 and 70 s) than in the case of control pairs. Unfortunately, we do not have any information about the offspring of these thelytokous females, or whether sperm transfer occurred during these matings. The duration of copulation, however, seems to correlate with the amount of sperm transfer in insects (e.g., [47,82]); therefore, it is regarded as a type of cryptic male mate choice, meaning that there is variation in the amount of resources allocated to females due to differences in their quality or strategic value for the males [79,83]. We think that the above outlined observations strengthen the opinion of Mendelson and Shaw [84], i.e., (potential) mating partners do not recognize each other on species level (compatibility) and value the mate quality independently of that. They claim that “compatibility is a subset of quality, representing one of many axes of variation in mate quality”, hence, “every mating decision is a mate quality decision” [84]. It follows from this that, in our experiments, only two onion thrips males assessed females from different lineages in the cross pairings to be of a high enough quality to copulate with them, but even these males substantially reduced the duration of their mating. Based solely on the observed behaviours, we hypothesize that, in the *T. tabaci* lineages, the males are in control of the duration of mating, since usually their behaviour changes at detectable levels prior to separation. However, of course it would be desirable to test this hypothesis under different conditions as well as with a higher number of replications.

Divergence among cross and control pairings in the *T. tabaci* cryptic species complex appears to start only after interaction, and become even stronger after male mounting, indicating the presence of cues acting only upon contact, such as CHCs. Communication in arthropods by CHCs seems to be widespread [22,23], and such substances have already been studied in thrips [85,86]. Despite the clear importance of CHCs in insect communication for mate recognition, there seems to be a discrepancy in the reliance solely on CHCs as sexual cues [24]. In addition, in a recent study, vibrational communication, even at close range, was found to be necessary to accomplish mating [25], but no study to date has investigated this type of communication in thrips. Tactile assessment [79] and simple behavioural cues provided by alive insects [24,81] could also be very important. Since our observations did not reveal any differences in the precopulatory or copulatory behaviour that could explain the lack of mating in the cross pairings, we assume that variation in the contact pheromones might be the cause of reproductive isolation among the lineages. Nonetheless, we suggest that further research should investigate the roles of CHCs as well as the possibility of vibrational communication in the onion thrips cryptic species complex.

Taking all these ideas together, regarding the species status of *T. tabaci* lineages, we can state the following. The most widely accepted concept for species is the biological species concept [16]; however, this is hard to apply for asexually reproducing organisms, such as for thelytokous thrips. Instead, concepts such as the evolutionary species concept [87] or a unified species concept (as proposed by de Queiroz [88]) fit better. Reproductive isolation, however, could mean the presence of a distinct species under either of these two concepts, too, as clear evidence of a “separately evolving metapopulation lineage” [88], which “maintains its identity from other such lineages” [87].

We demonstrated that mating occurs easily between specimens from the same lineage under laboratory conditions (if interaction occurred, more than 90% of the pairs from the T♀ + T♂ and L1♀ + L1♂ combinations mated); therefore, the lack of copulation in other treatments indicates reproductive isolation, which means that the two arrhenotokous lineages of onion thrips should be considered as distinct species. We believe that, despite the low number of heterospecific matings in L2♀ + T♂ combinations, these lineages also do not belong to the same species, because the no-choice design likely increased the chance of heterospecific matings, and the shorter copulation time likely caused a reduced sperm transfer. Earlier studies, which showed divergences in DNA sequences [57,63] and differences in morphometric variables [62] among onion thrips populations collected from different plants and locations, strengthen the hypothesis that the T lineage is a separately evolving lineage from either of the L lineages, and thus a distinct species. However, in order to clarify this question, further experiments are required in the future with the investigation of potentially hybrid progeny with molecular markers.

Note that, even if we hypothesize that the current reduction in (or lack of) gene flow among the T and L lineages of *T. tabaci* is due to differences in their close range communication, we still do not know the barrier(s) that originally initiated the speciation process in the past, as these are not necessarily the same as present barriers [16,89]. Host shift is likely to have played a role and led to the evolution of new isolating barriers to reproduction, but there is no evidence that host shift itself initiated speciation [90]. This is worth investigating in order to understand speciation in *T. tabaci* and its consequences for the differences in the biology of the lineages.

### 4.2. Mating Behaviour and Post-Mating Interactions

To our knowledge, this is the first comprehensive study of the reproductive behaviour of onion thrips that involved all three known lineages from this cryptic species complex. We conclude that there are no marked differences among lineages and that the copulation behaviour of *T. tabaci* lineages resembles that of other thripid species. Slight differences were detectable however, as outlined below, together with other important points.

Milne et al. [45] observed that, in *F. schultzei*, perhaps from a slightly longer distance than in our setup, it was almost always the female who approached the male. In contrast, in our experiments, such consistent behaviour was not detectable; usually, it was the male who approached the female in the interaction events that led to successful mating; however, frequencies differed across combinations. This variation indicates that likely no volatile pheromone is involved in the communication of individuals at such short distances, or at least they are not produced by lone individuals. Since earlier studies suggest that male patrolling and fighting behaviour might be necessary for the production of aggregation pheromones in other thripid species as well [91,92], it is not surprising, that under these experimental conditions we found no evidence for such substances in onion thrips lineages. In the T lineage (T♀ + T♂), however, where more than 90% of the pairs mated at their first interaction event, and in 80% of the cases, males approached the females, the possibility of a female-produced pheromone cannot be completely ruled out. A female-specific volatile substance is only known in *E. americanus*, but its function is not known yet [93]. Instead of supposing a female-produced pheromone, an alternative hypothesis could be simply a difference between sexes in their activity and exploratory behaviour. Either way, future investigations for both the presence of aggregation pheromones and for female-specific substances are needed.

Unlike in the case of *F. schultzei* and *S. aurantii* where the male responds to the proximity of the female with rapid movements of his antennae, either before first contact [45] or before he mounts the female [46], we observed just the opposite in all of the onion thrips lineages. Males in our experiments usually performed a sudden halt in their movements, antennae included, usually just before making contact with the female and mounting her. A similar behaviour was also described in *F. occidentalis* by Akinyemi [94]. We called this “identification behaviour”; however, neither the purpose, nor the kind of cues that are processed during this behaviour by the individuals are known yet.

The first contact position during the precopulation in *T. tabaci* seems to match the behaviour of *F. occidentalis* and *E. americanus*, including the observations that (1) this does not affect the success of copulation, (2) the females often show some kind of receptive behaviour (either lowering her abdomen and thorax or bending the tip of her abdomen up) and (3) the contact, the male mounting and the copulation usually follow each other rather quickly [43,44,47].

Antennation and stroking the females’ backs with the legs by the males seems to be a characteristic part of thrips species’ copulation behaviour, and this is also the case in the onion thrips cryptic species complex. Our observations seem to differ merely slightly from those detected by other authors in the case of *F. occidentalis* [43,44]. We found it difficult to determine which parts of the females’ body (antennae, head or thorax) were touched by the males’ antennae during precopulation and copulation, but we did not find that touching the antennae of the female (or the antennal bases) would be necessary for successful copulation, as was suggested by Terry and Schneider [43]. It is hard to determine if behaviours of males during (pre)copulation are part of a courtship behaviour, serve the purpose of assessment of female quality or both [79]. We observed that both the antennation and the stroking start in an excited manner, and calm down during the copulation itself; therefore, we speculate that these behaviours might be part of a “female quality assessment behaviour” through tactile cues, or might indeed serve to calm the female, even if perhaps not exactly in the same way as was proposed by Terry and Schneider [43].

For some thripid species, the formation of a so-called “V-position” by the copulating male and female seems to be common [41,45], while in the onion thrips cryptic species complex, we found that this position is not characteristic, and even if it happens, it seemed that it was not intentional, rather it happened by accident. Note that Akinyemi et al. [41] hypothesized that the stroking behaviour of males could be the method for applying the anti-aphrodisiac pheromone, which also seems plausible, and males cannot mark the females with this substance while in the V-position.

Our results revealed that the duration of copulation was shortest in the L1♀ + L1♂ pairings, with significantly more time spent in copula by the L2♀ + L1♂ and T♀ + T♂ pairs. Aside from the duration of copulation, we also measured the duration of precopulation; however, for this, we used three different but well-defined starting points. The reason for this was that, in the literature, we sometimes found either a lack of definition or inconsistency in the use of this term, which made comparisons among species difficult. Despite the fact that no statistical significance was found among the lineages, it was noticeable, that precopulation was again always the shortest in the L1♀ + L1♂, and longest in L2♀ + L1♂ pairings, irrespective of the starting point for measurement. Reproduction and the behaviours associated with it can carry an increased risk of predation [95]. While this is not always evident as a consequence of copulation itself [96], physically paired insects can suffer from a higher risk of attacks by natural enemies [97,98], likely as a result of greater visibility, together with reduced mobility and escape capacity [97,99]. Therefore, we assume that a shorter copulation time can have an evolutionary advantage in thrips as well, even though this has not been studied in thripid species yet. Interestingly, Luan et al. [13] pointed out that both the duration of precopulation and copulation are shorter in the more successful, invasive species of the *Bemisia tabaci* species complex, than in the indigenous species. While we do not believe that this trait alone could be responsible for evolutionary success, we note that, based on the data available, it seems that the L1 lineage onion thrips is indeed also more widespread in the world, than the T lineage.

Analysis of the post-mating interactions showed significant differences in the pairs’ behaviour before and after copulation. In the interaction events after mating, males’ willingness to mount the females was reduced, and even if they did so, an even-more-pronounced change in their behaviour was detectable, since in 76–88% of the cases (when male mounting occurred), males rejected the females, eventually climbing down from their back. We believe that these results strongly indicate the presence of an anti-aphrodisiac pheromone, which acts primarily upon contact, and is presumably produced by the males and applied to the females during copulation. While in thrips such substances are only identified so far in *E. americanus* [49], our findings and hypothesis also match the findings of Akinyemi and Kirk [44], who observed that experienced western flower thrips males mated with virgin females, but avoided copulating with mated females, and instead walked away sooner or later after contact with them.

In contrast with these observations, five pairs in total remated during our standard observation period. However, in four cases out of five, the duration of these second matings was substantially shorter than either the first mating of these pairs, or than the average duration of the first matings in general. The only exception was an L1♀ + L1♂ pair, whose first copulation was 109 s, and the second was 156 s long. A possible explanation for this case could be that the first mating was somehow ineffective, and thus repeated by the pair (a case that looks similar at first glance was also reported by Terry and Schneider [43]).

In *E. americanus*, while there is a decrease in the amount of male accessory gland material transferred to females, females receive a similar amount of spermatozoa from a male in successive copulations [47]. Therefore, no substantial differences would be expected in the duration of copulations of a male, as is seen indeed in *F. occidentalis*, where the duration of the successive matings of a male with different females did not differ significantly from each other [43]. Hence, this indicates that, in four cases out of five, remating males in our experiments likely detected the mating status of the female, and decided to mate with them, but only for a reduced amount of time, as a type of cryptic male mate choice. This hypothesis is supported by the observations of Reinhold et al. [83] with birch catkin bugs, where it became evident that, while males remated with the same female, the durations of these copulations were significantly shorter than those when males copulated with a novel (but still recently mated) female.

In T♀ + T♂ pairings, after copulation, there was a significant increase in the ratio of interaction events with rejection behaviour (after male mounting) by the females as well. The reason behind this is unknown, but it indicates that, in this species, both the females and the males try to avoid unnecessary rematings. Interestingly, in western flower thrips, Terry and Schneider [43] observed that mated females rejected subsequent males with flipping their abdomen up and down, while Akinyemi and Kirk [44] observed that the males avoided copulating with mated females without encountering any observable rejection behaviour from the females. In the T lineage of onion thrips, it seems that both could be relevant.

### 4.3. Mating of Thelytokous Females and Hybridization

Females in asexual lineages usually show a certain level of reduced function of sexual traits, though these are often traits not related to copulation itself, rather to mate location, attraction and fertilization of the eggs [100,101]. In Thysanoptera, however, the reduced size (*Parthenothrips dracaenae*; [102]) or complete loss (*Heliothrips haemorrhoidalis*; [103]) of the spermatheca is also known. The mating behaviour of the thelytokous *Franklinothrips vespiformis* appeared to be normal [104], while no copulation occurred in pairs of thelytokous females and arrhenotokous males of *Thrips nigropilosus*, despite males attempting to copulate [105]. Our results confirmed the results of Li et al. [73] for mating between thelytokous and arrhenotokous onion thrips; however, the mating of males from the L1 lineage was somewhat less frequent in pairings with females of the L2 lineage, than with L1 females, but this difference was not significant. Other observed traits, however, indicate slight differences in the behaviour of thelytokous and arrhenotokous females from the L lineages of the *T. tabaci* cryptic species complex.

Compared to the L1♀ + L1♂ combinations, a longer duration of precopulation and copulation was detected in L2♀ + L1♂ pairings, though the difference was only significant in the case of copulation. Based on this, we propose the hypothesis that, while males of the L1 lineage mate with the thelytokous females, these pairs might need more time to properly recognize and assess each other as mating partners. Moreover, either in the case of the first interaction event, or in the case of the interaction event that led to successful copulation in the L1♀ + L1♂ combinations, there was no statistical difference in the frequencies of male vs. female approaching, but in the L2♀ + L1♂ pairings, males approached the females significantly more often than vice versa. This indicates a higher rate of activity or exploratory behaviour of virgin L1 females, than of virgin thelytokous females, which might be explained by the greater necessity for arrhenotokous females to find a suitable mate.

Perhaps the most interesting differences were found in the rejection behaviour by females. Usually, females were calm in general in our experiments throughout mating in all the lineages, but we detected a rather high frequency of some kind of rejection behaviour during precopulation (arching and/or flipping their abdomen up and down). This was also reported by Akinyemi et al. [41] in *M. sjostedti*, and one of the possible explanations offered by those authors was that this behaviour might serve as a screening of suitable mates. While this hypothesis sounds conceivable, our results do not seem to support it, as rejection behaviour was the most frequent in the case of L2♀ + L1♂ pairings, and the rarest in the L1♀ + L1♂ combinations, and the difference was slightly significant. Since a thelytokous female does not necessarily need to mate in order to produce viable female progeny, and even if they mate, the rate of gene transfer is quite low [73], it is logical to assume the presence of a somewhat “stronger” screening for higher quality males in arrhenotokous females. Perhaps the more frequent rejection behaviour of thelytokous females is a consequence of difficulties for these females in recognizing approaching L1 males as mating partners, indicating some form of loss of function in the females’ ability to receive cues from males and assess their sexual traits.

Kobayashi et al. [74], after having performed microsatellite analysis at nine loci, claimed that sexual and asexual onion thrips lineages are genetically isolated. However, Li et al. [73] demonstrated that there is not just copulation between the arrhenotokous and thelytokous lineages (which is confirmed by our study), but there is also a low rate of gene transfer. This emphasizes the importance of mating studies as well as molecular investigations. It also questions the species status of L1 and L2 *T. tabaci* lineages. Considering, on the one hand, the evidence of gene transfer and the shared ecological niches, and on the other hand, the genetic differences together with the assumed reduced function in the sexual traits of thelytokous females, we believe that these two lineages are diverging from each other, and are likely currently in a so-called “gray zone” [88] in the speciation process, while depending on the applied species criteria, they can be viewed as either one or two species.

Brunner et al. [57] estimated that the divergence of the L1 and L2 lineages occurred approximately 21 million years ago. This would mean that the thelytokous onion thrips might be among the most ancient asexual insect lineages (see [106] for review); however, the lack of complete genetic isolation from the L1 lineage clearly aids the long-lasting existence of the otherwise asexually reproducing L2 lineage.

We did not test the rate of gene transfer from arrhenotokous males to daughters of thelytokous mothers but found an arrhenotokous *T. tabaci* isofemale line from our L1 culture harbouring an mtCOI characteristic of the L2 lineage, and we believe this also confirms the possibility of gene transfer between L1 and L2 lineages. To our knowledge, this is the first report of such “hybrids” from Europe. Earlier, however, in Japan, Sogo et al. [68] and Aizawa et al. [107] also found strains harbouring mtCOI sequences that place them in the monophyletic thelytokous group, and this means that the gene flow among these lineages might be frequent in nature.

We did not include the pairs (three pairs in total) with males from this “hybrid” isofemale line in the statistical analysis, because the mtCOI and the observed reproductive mode were contradictory. The analysis of these males either together with the other L1 males, or separately (as “L2 males”) would be a mistake. However, we observed that one of these males confined with an L1 female, interestingly, did not copulate with her during the 10-min-long observation period. Even though they contacted each other, the female seemed receptive and stood still. Although the male mounted her, performed antennation and stroking behaviour and bent his abdomen, he was still not able to insert his aedeagus into the female genitalia. Since, on the next occasion, he did not align his body to face the same direction as the female, the female eventually started to reject him and run away without copulation. (The two remaining males from this isofemale line did not interact with the females during the 10-min-long observation period.) We cannot rule out the possibility, that even if gene flow occurs frequently, the fitness of these “hybrids” is reduced, but this needs further investigation.

### 4.4. Activity of the Lineages

Arthropod species and populations that differ from each other in their level of adaptation to urban environments, in their success as invaders or in their susceptibility to insecticides also show differences in their behavioural traits, such as activity, boldness and exploratory behaviour [108,109,110], implying that differences in these latter traits might have effects on the former traits. Though this clearly shows the importance of this topic, studies on thrips focusing on these behavioural traits are extremely rare. Riefler and Koschier [111] listed the behavioural repertoire of *T. tabaci* and measured the time spent with different behaviours—including inactivity and exploration—but the study was only focused on the thelytokous females. Reitz et al. [112], however, found that differences in the mobility of two species of thrips prey can have an important impact on the success of a generalist predator. The primary aim of our experimental design was not investigation of the insects’ activity, locomotion or other such behavioural traits. Interestingly though, our results still revealed certain differences among the lineages, which are likely the consequence of divergences in behavioural traits not necessarily linked solely to mating.

In our experiments, we were able to detect specific changes in the behaviour of individuals only if they were approximately 1–2 mm from each other. This is supported by the observations of Kirk [39], who also found the same in the case of *Thrips major* and *T. fuscipennis*, under natural conditions. Anthophilous thrips only turn towards small food items (pollen grains) when they are within a millimetre of them [113]. If thrips individuals do not sense each other when they are placed in an arena with an approximate diameter of 8.5 mm, then some of our results might only be explained as a consequence of differences in the activity and exploratory behaviour among lineages.

There was no significant difference across the six combinations in the time required for the first interaction event to occur. However, an analysis of which sex approached the other in the first interaction event showed notable differences, and these patterns do not seem to indicate the presence of any kind of cue that would make the thrips either search actively for, or try to avoid each other, instead they might only show variation in the exploratory behaviour. From such a point of view, it seems, that the T lineage has a substantially lower activity than the L lineages, since in the cross pairings, irrespective of sex, it was always the T lineage that approached the other individual fewer times. Our results also show that females from the L1 lineage have a higher activity than females from either the thelytokous (L2) or the T lineage. This was shown by the higher frequency of L1 females that approached males, together with results of the number of interaction events from the cross combinations.

We often observed that individuals in the cross pairings became very excited and started to run fast in the arena after one or more interaction events, and this behaviour could lead easily to ten, or even more, interaction events (Category 5) during the 10-min-long observation period. Category 5 was significantly more frequent in L1♀ + T♂ combinations, than in T♀ + L1♂, indicating that L1 females might get “irritated” more easily, resulting in more frequent encounters with the other individual in the arena. Therefore, in the L1♀ + T♂ combinations, more than 34% of the pairs interacted with each other at least ten times, while “no interaction event” was quite rare. In the cross pairings with the least active female (T♀ + L1♂), just the opposite was true both for Categories 1 and 5, while L2♀ + T♂ combinations were intermediate.

We found one more interesting phenomenon and difference between lineages, namely the escape responses of thrips at the end of the interaction events in the cross pairings. We observed that, in many cases, specimens from these pairings reacted to a contact with the other thrips by running away in excitement, usually with a rapid change in behaviour. Previously, Isenhour and Yeargan [114] found running to be the primary escape tactic of soybean thrips encountered by predatory bugs.

However, it is very difficult to explain why a thrips would try to escape from another phytophagous thrips, even if they do not recognize each other as suitable mating partners. Additionally, even if they did so, why would individuals from the T lineage exhibit an escape response more often than thrips from the L1 lineage, showing a notable difference in the behaviour of lineages? One might think that perhaps the differences in body size can explain this phenomenon, since, taking all the three cross combinations together, males responded with escape more often than females. Additionally, our personal observations suggested that individuals from the T lineage are smaller than thrips from the L lineages. However, in the T♀ + L1♂ pairings, females tried to escape more often, which goes against the hypothesis outlined above, because L1 males still seem to be smaller than T females. Note that in a recent study, González-Orellana et al. [115] found that *Liothrips jatrophae* (Thysanoptera: Phlaeothripidae) individuals exhibited an escape response after being exposed to extracts from conspecifics, indicating that extract compounds might function as an alarm signal. We believe that perhaps the investigation of contact pheromones of onion thrips lineages might help us reveal compounds not only responsible for mate recognition and assessment but will also aid the identification of semiochemicals that can trigger an escape response.

## 5. Conclusions

Our study revealed that the T and L lineages of the *T. tabaci* cryptic species complex are reproductively isolated from each other, and thus represent distinct species. Behavioural patterns of control (conspecific) and cross (heterospecific) pairs started to differ substantially only after contact, suggesting an important role of contact pheromones in assessing each other as suitable mating partners. In the control pairs, precopulatory and copulatory behaviour were almost identical among lineages, and these also resembled the mating sequence of other thripid species. A change in the behaviour of males after copulation indicated the existence of an anti-aphrodisiac pheromone, which is presumably applied to the females during copulation. We confirmed that onion thrips from the L1 and L2 lineages mate with each other; however, the observed differences point to some form of loss of function in the sexual traits of thelytokous females. Our results also showed that onion thrips lineages differ from each other in their level of activity and reaction upon contact with a heterospecific thrips. 

## Figures and Tables

**Table 1:** Mating behaviour sequence in the *Thrips tabaci* species complex.

Steps of the Mating Behaviour Sequence	Description
Phase 1: Interaction	The specimens are close enough (approximately 1 mm) to sense each other, and at least one of them shows an obvious change in behaviour.
Phase 2: Contact	Physical contact, irrespective of which specimen touched the other.
Phase 3: Male mounting	Male starts to climb on the back of the female.
Phase 4: Bend abdomen	Male curls his abdomen beneath that of the female.
Phase 5: Successful mating	Prolonged contact between the end of the male and female abdomen.

**Table 2:** List and description of monitored behaviour parameters during interactions of male and female thrips.

Behaviour Parameters Observed	Description
Interaction event	The specimens are close enough (approximately 1 mm) to sense each other, and at least one of them shows an obvious change in behaviour, but they do not necessarily contact physically, although the interaction event often starts with physical contact. The end of a given interaction event is when the specimens depart from each other for more than 2 mm without an obvious sign of looking after each other, or they might stay closer but without any sign of sensing each other.
The sex of the approaching specimen	The sex of the thrips that approached the other at the start of a given interaction event.
Contact position	Position of male and female during initial contact (head to head, female head to male thorax or abdomen, male head to female thorax or abdomen).
Female rejection	Female arching and/or flipping her abdomen up and down when a male contacts or mounts her.
Male rejection	Male starts to climb on the back of the female, but he stops later and discontinues mating with the female, and either reverses the movement immediately, or climbs down from the female’s back a few seconds later.
Escape response	At the end of a given interaction event, the specimen leaves the area in an excited manner (for example by running away), usually with a rapid change in behaviour.
Male identification behaviour	In close proximity to the female, for a brief period of time movement of any body part of the male is paused, including walking or running and moving the antennae, which are usually held fixed more or less high.

**Table 3:** The percentage of pairs that completed each step in the mating behaviour sequence for T-T, L1-L1 or L2-L1 *Thrips tabaci* pairs together with the significance level of their comparisons.

Step in the Mating Behaviour Sequence	T♀ + T♂			L1♀ + L1♂			L2♀ + L1♂		
	Both Specimens Used for the First Time	One or Both Specimens Used for the Second Time	*p* Value	Both Specimens Used for the First Time	One or Both Specimens Used for the Second Time	*p* Value	Both Specimens Used for the First Time	One or Both Specimens Used for the Second Time	*p* Value
Interaction	75%	50%	=0.128	90%	70%	=0.212	80%	77%	=0.572
	(*n* = 12)	(*n* = 30)		(*n* = 10)	(*n* = 23)		(*n* = 15)	(*n* = 26)	
Successful mating	67%	47%	=0.204	80%	65%	=0.339	53%	54%	=0.614
	(*n* = 12)	(*n* = 30)		(*n* = 10)	(*n* = 23)		(*n* = 15)	(*n* = 26)	
Successful mating (if interaction occurred)	89%	93%	=0.620	89%	94%	=0.600	67%	70%	=0.573
	(*n* = 9)	(*n* = 15)		(*n* = 9)	(*n* = 16)		(*n* = 12)	(*n* = 20)	

*p* values were calculated using Fisher’s exact test for a 2 × 2 contingency table with the frequencies of pairs that did or did not complete a step in the mating behaviour sequence against whether both individuals were used for the first time or one or both individuals were used for the second time.

**Table 4:** The percentage of pairs that completed each step in the mating behaviour sequence as a proportion of those (*n*) that completed the previous step.

Step in the Mating Behaviour Sequence	T♀ + T♂	T♀ + L1♂	L1♀ + L1♂	L1♀ + T♂	L2♀ + L1♂	L2♀ + T♂	*p* Value
Interaction	57%	67%	76%	94%	78%	78%	<0.010
	(*n* = 42)	(*n* = 45)	(*n* = 33)	(*n* = 32)	(*n* = 41)	(*n* = 41)	
	*a*	*ab*	*ab*	*b*	*ab*	*ab*	
Contact	96%	97%	92%	93%	88%	97%	=0.720
	(*n* = 24)	(*n* = 30)	(*n* = 25)	(*n* = 30)	(*n* = 32)	(*n* = 32)	
Male mounting	96%	17%	100%	36%	93%	29%	<0.001
	(*n* = 23)	(*n* = 29)	(*n* = 23)	(*n* = 28)	(*n* = 28)	(*n* = 31)	
	*b*	*a*	*b*	*a*	*b*	*a*	
Bend abdomen	100%	20%	100%	20%	96%	22%	<0.001
	(*n* = 22)	(*n* = 5)	(*n* = 23)	(*n* = 10)	(*n* = 26)	(*n* = 9)	
	*b*	*a*	*b*	*a*	*b*	*a*	
Successful mating ^1^	100%	0%	100%	0%	88%	100%	=0.103
	(*n* = 22)	(*n* = 1)	(*n* = 23)	(*n* = 2)	(*n* = 25)	(*n* = 2)	
Remating ^2^	14%	N.A.	9%	N.A.	0%	0%	N.A.
	(*n* = 22)		(*n* = 23)		(*n* = 22)	(*n* = 2)	

*p* values were calculated using Fisher’s exact tests for a 6 × 2 contingency table with the frequencies of pairs that did or did not complete a step in the mating behaviour sequence as one factor, and the six different pairings as the other. Different letters in italic within each row across columns show significant deviations between the percentages of different pairs that completed a step in the mating behaviour sequence (Marascuilo’s procedure, *p* < 0.05). ^1^ Exception is “Successful mating”, for which only T♀ + T♂, L1♀ + L1♂ and L2♀ + L1♂ pairings were included in the analysis; therefore, *p* values were calculated using Fisher’s exact test for a 3 × 2 contingency table. ^2^ No statistical analysis was conducted for “Remating”.

**Table 5:** The mean time (±SD) until the first interaction and the mean time (±SD) between the first contact and first male mounting.

	T♀ + T♂	T♀ + L1♂	L1♀ + L1♂	L1♀ + T♂	L2♀ + L1♂	L2♀ + T♂	*p* Value
Time until first interaction event (s)	110.75 ± 131.88	149.43 ± 157.57	90.20 ± 107.85	98.43 ± 127.62	97.59 ± 130.37	80.09 ± 91.61	=0.677
	(*n* = 24)	(*n* = 30)	(*n* = 25)	(*n* = 30)	(*n* = 32)	(*n* = 32)	
Time between first contact and first male mounting (s)	4.95 ± 12.42	157.60 ± 88.63	52.70 ± 144.01	168.50 ± 183.70	14.62 ± 29.73	113.78 ± 175.82	<0.001
	(*n* = 22)	(*n* = 5)	(*n* = 23)	(*n* = 10)	(*n* = 26)	(*n* = 9)	
	*a*	*c*	*ab*	*bc*	*ab*	*bc*	

*p* values were calculated using one-way ANOVA tests. Different letters in italics within each row across columns show significant pairwise differences between the pairings (Games-Howell post hoc test, *p* < 0.05).

**Table 6:** Percentage of cases when the male or the female approached the other thrips, or both of them were moving to the other in the first interaction event for the six pairings.

	Which Sex Approached			*p* Values of Comparisons		
	Male Was Approaching	Female Was Approaching	Both Were Approaching	Male vs. Female	Male vs. Both	Female vs. Both
T♀ + T♂	76.5%	23.5%	0.0%	<0.050	<0.001	=0.125
(*n* = 17)	*b*	*a*	*a*			
T♀ + L1♂	71.4%	17.9%	10.7%	<0.010	<0.001	=0.727
(*n* = 28)	*b*	*a*	*a*			
L1♀ + L1♂	42.9%	52.4%	4.8%	=0.824	<0.050	<0.010
(*n* = 21)	*b*	*b*	*a*			
L1♀ + T♂	14.3%	75.0%	10.7%	<0.010	>0.999	<0.001
(*n* = 28)	*a*	*b*	*a*			
L2♀ + L1♂	73.1%	19.2%	7.7%	<0.010	<0.001	=0.453
(*n* = 26)	*b*	*a*	*a*			
L2♀ + T♂	23.3%	66.7%	10.0%	<0.050	=0.344	<0.001
(*n* = 30)	*a*	*b*	*a*			

*p* values were calculated in each pairing using McNemar’s tests, comparing the number of cases when the male, the female or both were approaching. Different letters in italics within each row show significant differences between the possible scenarios within pairings (McNemar’s test, *p* < 0.05).

**Table 7:** Percentage of cases and the adjusted standardized residuals when the male or the female approached the other thrips, or both of them were moving towards the other in the interaction event that led to successful mating.

		Which Approached			*p* Values of Comparisons		
		Male Was Approaching	Female Was Approaching	Both Were Approaching	Male vs. Female	Male vs. Both	Female vs. Both
T♀ + T♂ (*n* = 15)	Percentage	80.0%	13.3%	6.7%	<0.050	<0.010	>0.999
		*b*	*a*	*a*			
	Adj. residuals	1.4	−1.5	−0.1			
L1♀ + L1♂ (*n* = 20)	Percentage	50.0%	45.0%	5.0%	>0.999	<0.050	<0.050
		*b*	*b*	*a*			
	Adj. residuals	1.7	2.2 *	−0.5			
L2♀ + L1♂ (*n* = 19)	Percentage	68.4%	21.1%	10.5%	<0.050	<0.010	=0.687
		*b*	*a*	*a*			
	Adj. residuals	0.4	−0.8	0.6			

*p* values were calculated in each pairing using McNemar’s tests comparing the number of cases when the male, the female or both were approaching. Different letters in italics within each row show significant differences between the possible scenarios within pairings (McNemar’s test, *p* < 0.05). Fisher’s exact test for a 3 × 3 contingency table was not significant (*p* = 0.250). * Significant at *p* < 0.05.

**Table 8:** Percentage of cases when males exhibited identification behaviour during precopulation.

T♀ + T♂ (*n* = 21)		L1♀ + L1♂ (*n* = 20)		L2♀ + L1♂ (*n* = 19)	
Percentage	*p* Value (One-Sample z-Test)	Percentage	*p* Value (One-Sample z-Test)	Percentage	*p* Value (One-Sample z-Test)
95.2%	<0.001	80%	<0.050	89.5%	<0.010

**Table 9:** Percentage of the first contact positions during the precopulation.

	Contact Position			*p* Values of Comparisons		
	Head to Head	Male Head to Female Thorax or Abdomen	Female Head to Male Thorax or Abdomen	Male Head to Female vs. Female Head to Male	Male Head to Female vs. Head to Head	Female Head to Male vs. Head to Head
T♀ + T♂ (*n* = 22)	45.5%	54.5%	0.0%	<0.001	=0.832	<0.001
	*b*	*b*	*a*			
L1♀ + L1♂ (*n* = 23)	47.8%	39.1%	13.0%	=0.146	=0.824	=0.057
	*a*	*a*	*a*			
L2♀ + L1♂ (*n* = 22)	40.9%	40.9%	18.2%	=0.267	>0.999	=0.267
	*a*	*a*	*a*			

*p* values were calculated using McNemar’s tests comparing the number of cases for different contact positions in each pairing. Different letters in italic within each row show significant differences between the possible scenarios within pairings (McNemar’s test, *p* < 0.05).

**Table 10:** Percentage of cases when female rejection occurred during the precopulation and the adjusted standardized residuals.

		Rejected
T♀ + T♂ (*n* = 22)	Percentage	40.9%
	Adj. residuals	−0.10
L1♀ + L1♂ (*n* = 23)	Percentage	26.1%
	Adj. residuals	−1.88 ^+^
L2♀ + L1♂ (*n* = 22)	Percentage	59.1%
	Adj. residuals	2.01 *

^+^ Slightly significant at *p* = 0.06; * Significant at *p* < 0.05.

**Table 11:** The mean duration (±SD) of precopulation and copulation (s).

Mean Duration of	T♀ + T♂	L1♀ + L1♂	L2♀ + L1♂	df	F	*p* Value
	(*n* = 21)	(*n* = 21)	(*n* = 21)			
Precopulation 1	15.67 ± 13.15	13.05 ± 7.22	16.29 ± 9.42	2;60	0.637	=0.532
Precopulation 2	11.10 ± 9.91	10.19 ± 4.95	14.86 ± 9.66	2;60	1.510	=0.229
Precopulation 3	7.71 ± 10.00	5.67 ± 3.62	9.57 ± 8.63	2;60	1.650	=0.201
Copulation	176.10 ± 26.02	140.43 ± 16.64	155.57 ± 13.90	2;60	18.194	<0.001
	*c*	*a*	*b*			

*p* values were calculated using one-way ANOVA tests with Bonferroni’s correction. Different letters in italics within each row show significant pairwise differences between the pairings (Games-Howell test: *p* < 0.01).

**Table 12:** The mean ratio of interaction events with male mounting, male rejection and female rejection, analysed before and after copulation.

	T♀ + T♂			L1♀ + L1♂			L2♀ + L1♂		
Mean Ratio of Interaction Events with	Before Copulation	After Copulation	*p* Value	Before Copulation	After Copulation	*p* Value	Before Copulation	After Copulation	*p* Value
Male mounting ^1^	0.94	0.65	<0.001	0.81	0.47	<0.001	0.89	0.75	=0.169
	(*n* = 22)	(*n* = 20)		(*n* = 23)	(*n* = 15)		(*n* = 22)	(*n* = 22)	
Male rejection ^2^	0.05	0.76	<0.001	0.00	0.81	<0.001	0.05	0.88	<0.001
	(*n* = 22)	(*n* = 20)		(*n* = 23)	(*n* = 13)		(*n* = 22)	(*n* = 22)	
Female rejection ^2^	0.41	0.87	<0.001	0.28	0.29	=0.844	0.45	0.69	=0.107
	(*n* = 22)	(*n* = 20)		(*n* = 23)	(*n* = 13)		(*n* = 22)	(*n* = 22)	

*p* values were calculated with paired Wilcoxon’s signed rank tests. ^1^ The ratio of interaction events with male mounting was calculated as the number of interaction events when male mounting occurred divided by the total number of interaction events. ^2^ The ratio of interaction events with male or female rejection was calculated as the number of interaction events when male or female rejection occurred after male mounting divided by the number of interaction events when male mounting occurred.

**Table 13:** Percentage of replicates in categories 1–5 based on the total number of interaction events and the adjusted standardized residuals.

		Category 1:	Category 2:	Category 3:	Category 4:	Category 5:
		0 Interaction Event	1 Interaction Event	2–6 Interaction Events	7–9 Interaction Events	10 or More Interaction Events
T♀ + L1♂ (*n* = 45)	Percentage	33.3%	20.0%	31.1%	8.9%	6.7%
	Adj. residuals	2.2 *	1.1	−0.8	0.1	−2.5 *
L1♀ + T♂ (*n* = 32)	Percentage	6.3%	9.4%	46.9%	3.1%	34.4%
	Adj. residuals	−2.6 **	−1.1	1.6	−1.3	2.8 **
L2♀ + T♂ (*n* = 39)	Percentage	23.1%	15.4%	30.8%	12.8%	17.9%
	Adj. residuals	0.1	<0.1	−0.7	1.1	<0.1

Fisher’s exact test for a 5 × 3 contingency table was significant (*p* = 0.012). * Significant at *p* < 0.05; ** Significant at *p* < 0.01.

**Table 14:** Percentage of interaction events when females or males exhibited an escape response at the end of the interaction event and the adjusted standardized residuals.

		T♀ + L1♂		L1♀ + T♂		L2♀ + T♂		*p* Value
		Escape Response	No Escape Response	Escape Response	No Escape Response	Escape Response	No Escape Response	
Females	Percentage	36.8%	63.2%	8.4%	91.6%	18.3%	81.6%	<0.001
		(*n* = 35)	(*n* = 60)	(*n* = 12)	(*n* = 131)	(*n* = 22)	(*n* = 98)	
	Adj. residuals	5.1 ***	−5.1 ***	−4.3 ***	4.3 ***	−0.3	0.3	
Males	Percentage	28.7%	71.3%	55.9%	44.1%	37.5%	62.5%	<0.001
		(*n* = 27)	(*n* = 67)	(*n* = 80)	(*n* = 63)	(*n* = 45)	(*n* = 75)	
	Adj. residuals	−3.2 **	3.2 **	4.2 ***	−4.2 ***	−1.4	1.4	

*p* values were calculated using Fisher’s exact test for a 3 × 2 contingency table with the frequencies of interaction events in which a thrips did or did not escape at the end of the event against the cross-mating pairs. Separate analysis was carried out for males and females with the same method: “*n*” means the number of cases falling into each category. ** Significant at *p* < 0.01; *** Significant at *p* < 0.001.

## Data Availability

The data presented in this study are available on request from the corresponding author.

## Supplementary Materials

The following supporting information can be downloaded at: https://www.mdpi.com/article/10.3390/biology11030396/s1, Video S1: Copulatory behaviour of *Thrips tabaci* specimens from the L1 lineage; Video S2: Copulatory behaviour of *Thrips tabaci* specimens from the T lineage; Video S3: Copulatory behaviour of *Thrips tabaci* specimens (female from the L2, male from the L1 lineage); Video S4: An interaction event of *Thrips tabaci* specimens (female from the T, male from the L1 lineage) with an escape response of the female; Video S5: An interaction event of *Thrips tabaci* specimens (female from the L1, male from the T lineage) with an escape response of the male; Video S6: An interaction event of *Thrips tabaci* specimens (female from the L2, male from the T lineage) without an escape response; Video S7: Post-mating rejection behaviour (already mated *Thrips tabaci* specimens from the T lineage).
